# Supplementary material for: Global protein turnover quantification in Escherichia coli reveals cytoplasmic recycling under nitrogen limitation
Source: Nat Commun. 2024 Jul 13;15:5890. doi: 10.1038/s41467-024-49920-8 (PMC11246515; doi:10.1038/s41467-024-49920-8)
Supplement: Supplementary file 1 — Supplementary Information [file 41467_2024_49920_MOESM1_ESM.pdf]

Supplementary Figures

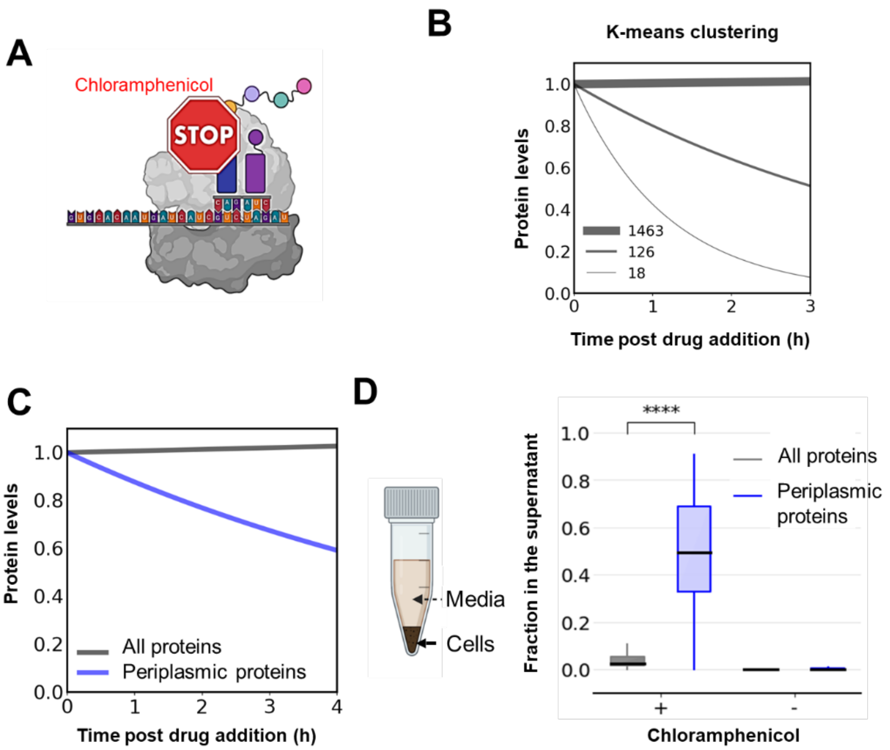

Supplementary Figure 1. Using translation inhibition to measure protein turnover might lead to unintended side effects. A) Protein synthesis is halted by adding chloramphenicol to a culture of exponentially dividing E. *coli* cells. Proteomic analysis samples are collected post-drug addition, with time points fitted to an exponential decay curve using half-life as a parameter. **B-C)** The shown curves are obtained using the fitted half-lives. **B)** k-means clustered protein abundance profiles after the addition of drug. The largest cluster of proteins do not change abundance, indicating that they are stable. **C)** Median protein levels over time for all proteins and periplasmic proteins. The median periplasmic protein appears to be degrading, while most of the proteins are stable. **D)** We separated the supernatant from the cells using centrifugation and quantified relative protein levels in both samples with proteomics. We calculated the fraction in the supernatant for each protein by dividing the abundance in the supernatant by the total abundance for that protein. Our data indicate that the supernatant is enriched with periplasmic proteins following chloramphenicol addition, suggesting that chloramphenicol may prompt the release of periplasmic proteins into the supernatant. Created with Biorender[1]

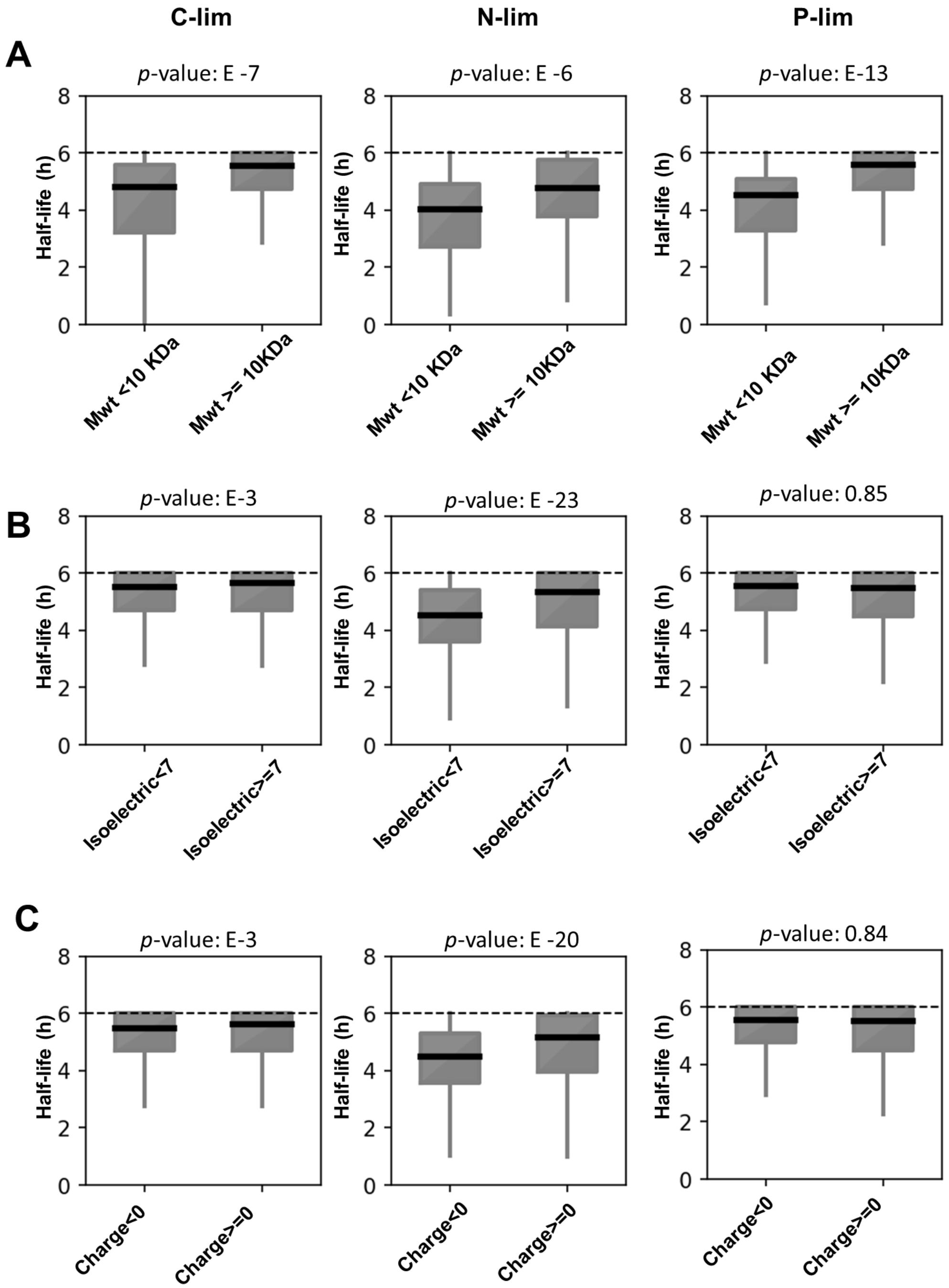

Supplementary Figure 2. Relationship between protein half-life and their physiochemical properties. A) Box plots of protein half-lives categorized by molecular weight for C, N, and P-lim conditions. Notably, smaller proteins exhibit significantly shorter half-lives. **B)** Box plots of protein half-lives categorized by isoelectric points for C, N, and P-lim conditions. Under N-lim, shorter-lived proteins are significantly more acidic, while no significant effect is observed under C and P-lim. **C)** Box plots for half-lives of proteins separated by their charge for C, N, and P-lim. Shorter lived proteins tend to be negatively charged under N-lim, However, there is no significant effect under C and P-lim. (p-values are obtained using a one sided Mann-Whitney U)

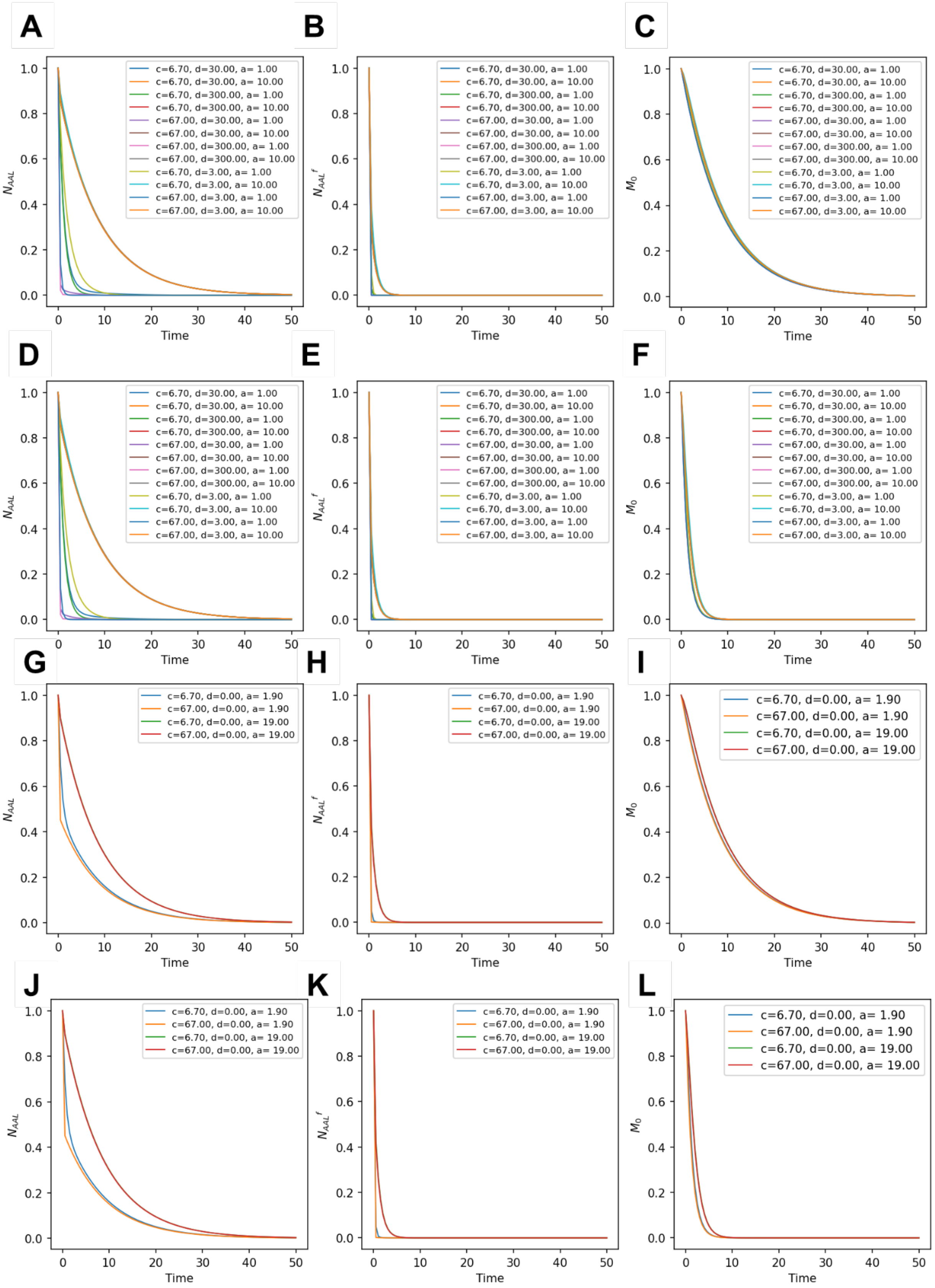
A
B
C
D
E
F
G
H
I
J
K
L
N_{AAL}
$N_{AAL}{}^{f}$
M_0
Time
c=6.70, d=30.00, a= 1.00
c=6.70, d=30.00, a= 10.00
c=6.70, d=300.00, a= 1.00
c=6.70, d=300.00, a= 10.00
c=67.00, d=30.00, a= 1.00
c=67.00, d=30.00, a= 10.00
c=67.00, d=300.00, a= 1.00
c=67.00, d=300.00, a= 10.00
c=6.70, d=3.00, a= 1.00
c=6.70, d=3.00, a= 10.00
c=67.00, d=3.00, a= 1.00
c=67.00, d=3.00, a= 10.00
c=6.70, d=0.00, a= 1.90
c=67.00, d=0.00, a= 1.90
c=6.70, d=0.00, a= 19.00
c=67.00, d=0.00, a= 19.00

Supplementary Figure 3. Plots of N_{AAL}, N_{AAL}^{f}, M_0 with time for range of parameter values

A-C) Case 1: Model P-lim/C-lim such that $N_V \neq 0$, for a peptide which is not actively degrading such that :-> k_D=0, f = 8, and D= log (2)/6

D-F) Case 2: Model P-lim/C-lim such that $N_V \neq 0$, for a peptide which is actively degrading with the active half-life of 1 hour, such that :-> k_D=log (2)/1, f = 8, and D= log (2)/6

G-I) Case 3: Model N-lim such that $N_V = 0$, for a peptide which is only diluting such that :-> k_D=0, f = 8, and D= log (2)/6

J-L) Case 4: Model N-lim such that $N_V = 0$, for a peptide which is actively degrading such that :-> k_D=log (2)/1, f = 8, and D= log (2)/6

28

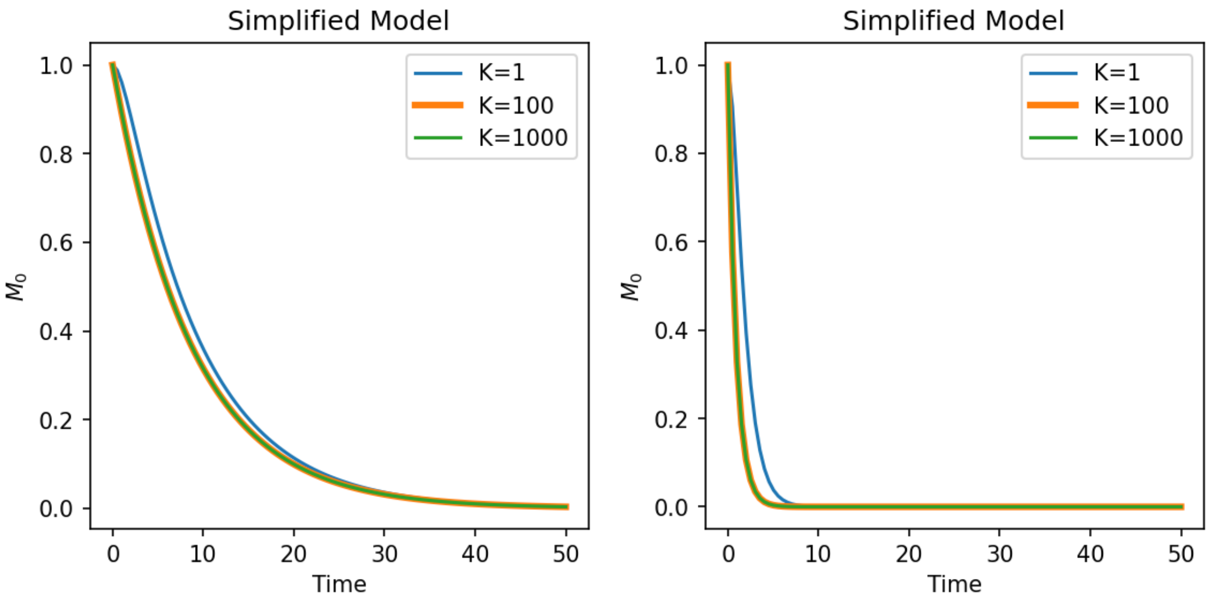

Supplementary Figure 4. Sensitivity analysis for the simplified model on parameter K. The plots show the profiles of M_0 with time for range of parameter values. The number of nitrogens f=8 and D= log (2)/6. Left) The dilution-only curve such that the active degradation rate k_D=0. Right) The curves when the active degradation rate is k_D=log (2)/1.

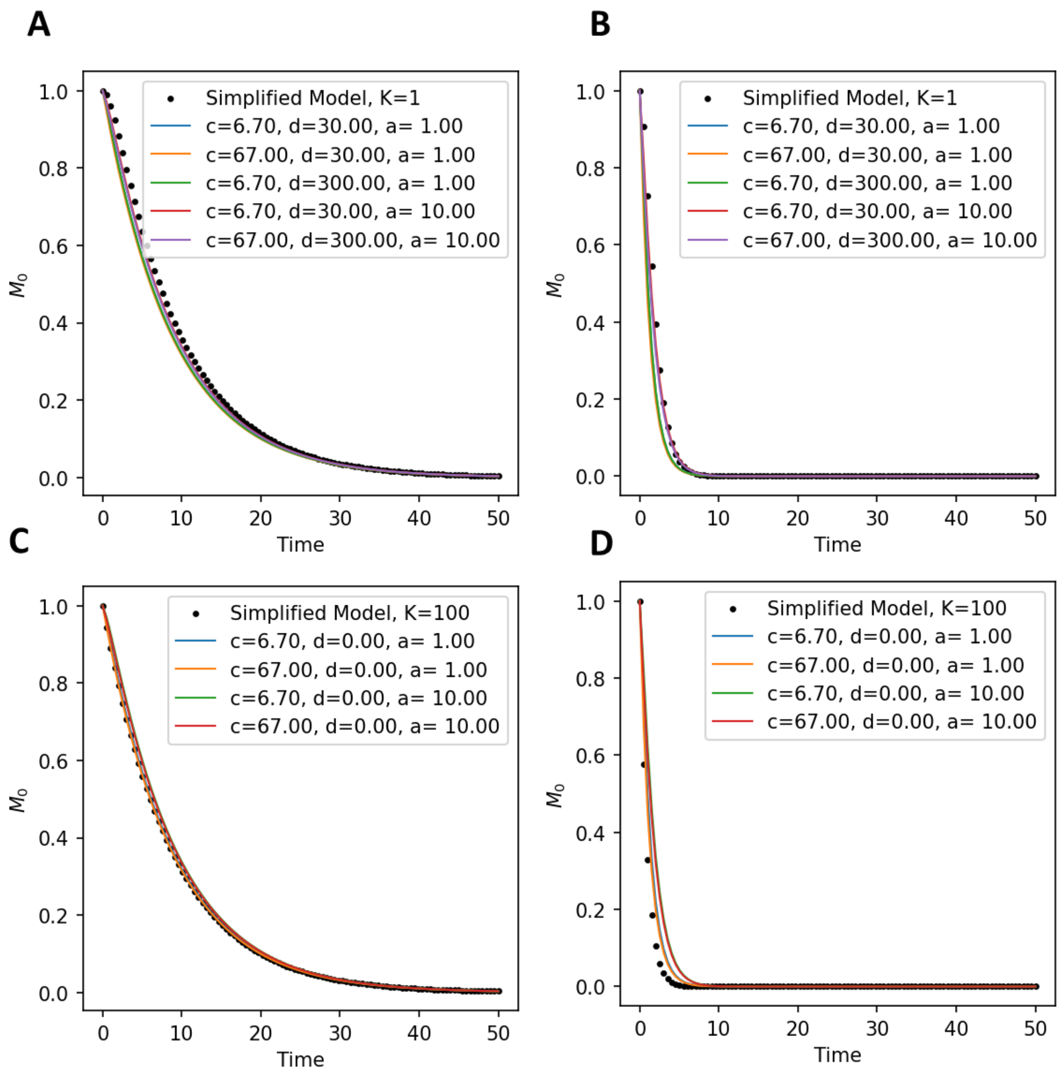

Supplementary Figure 5. Demonstration of equivalence of the detailed and simplified model by comparing M_0 relative levels plotted with time. The number of nitrogens f=8 and D= log (2)/6

A) Dilution-only curve such that the active degradation rate k_D=0 for P-lim and C-lim.
B) k_D=log(2)/1 for P-lim and C-lim
C) Dilution-only curve such that the active degradation rate k_D=0 for N-lim
D) k_D=log(2)/1 for N-lim

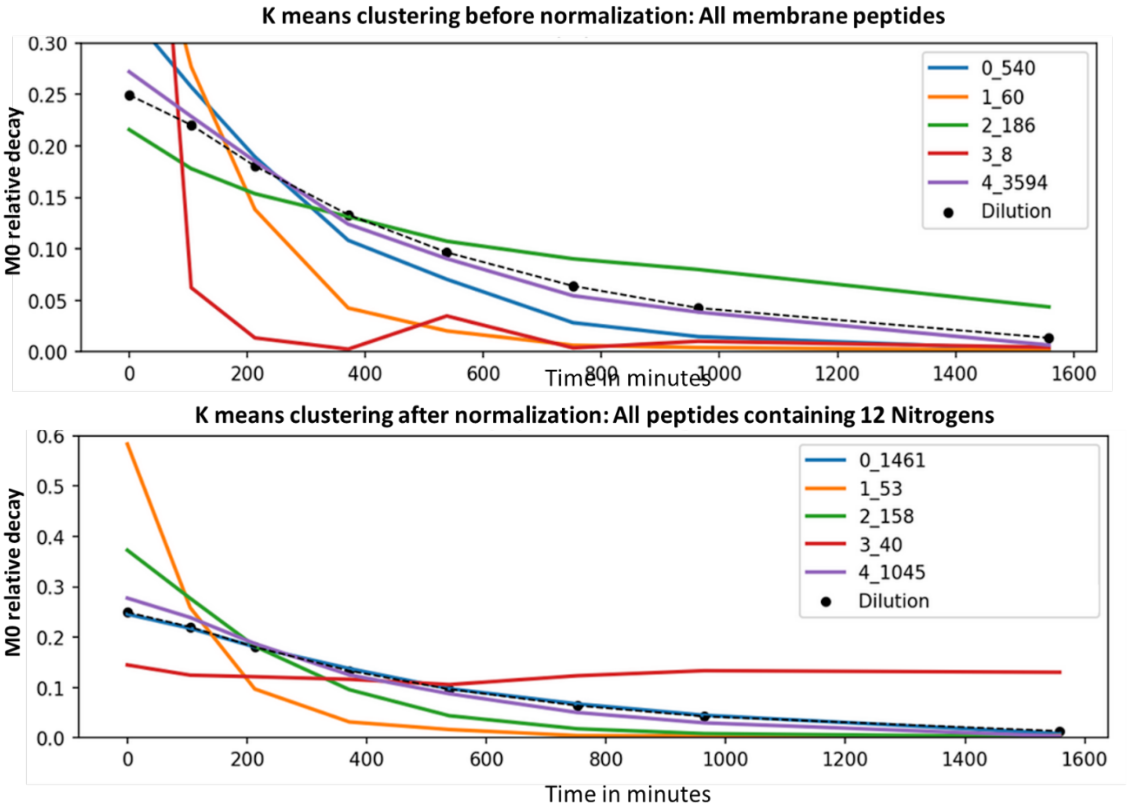

Supplementary Figure 6. Clusters of M_0 relative levels plotted with time. Clustering is done using k means algorithm. The dotted black curve is the dilution curve. The numbers in the legend correspond to the number of peptides in a cluster. Top) K means clustering before normalization for all the membrane peptides. Bottom) K means clustering after normalization for all the peptides containing 12 nitrogens.

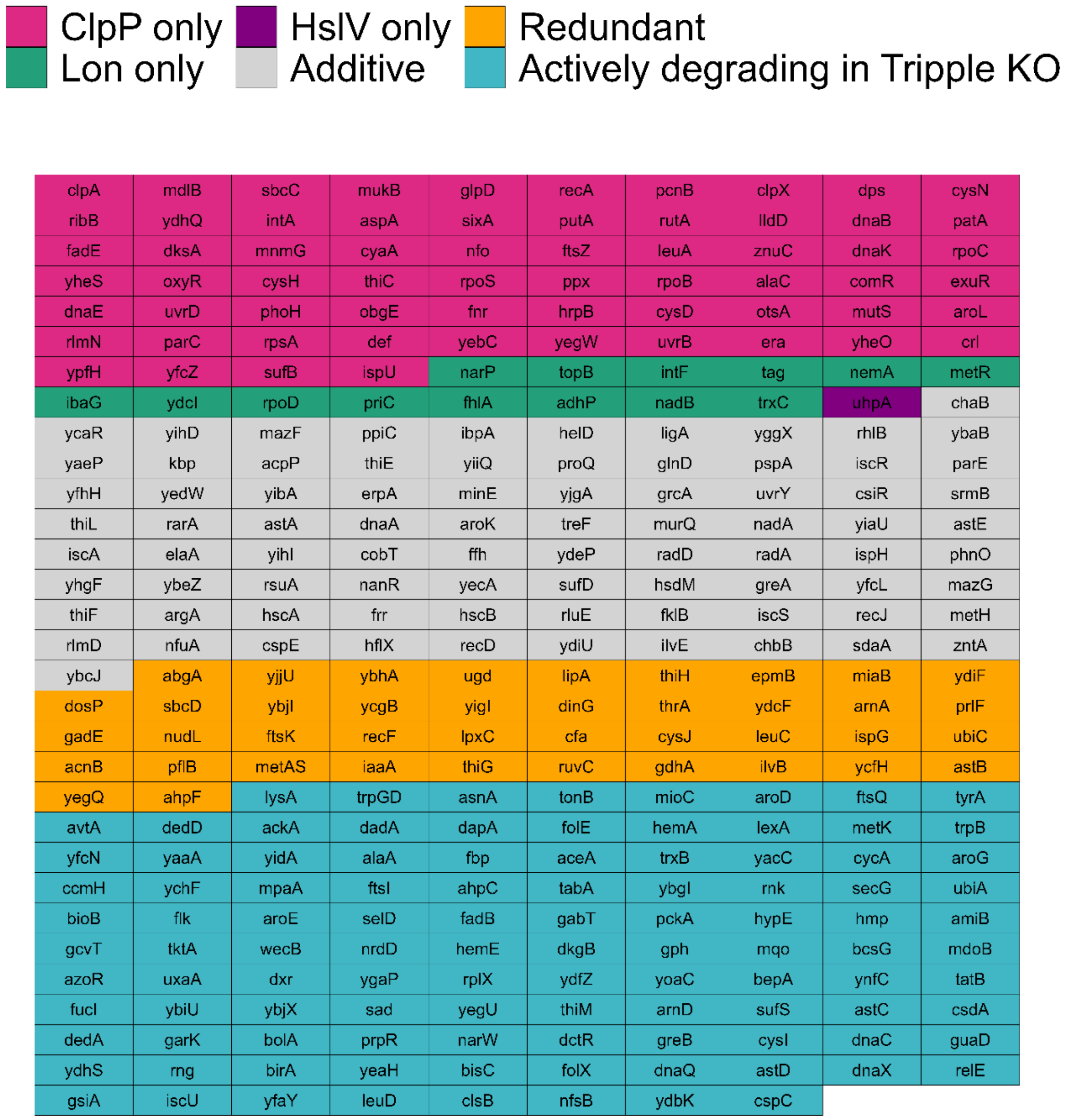

Supplementary Figure 7. Protease substrate assignments. We assign each protease's contribution to active protein turnover of the rapidly degrading proteins, as in Figure 3. Each protein was then assigned to one of six categories as described in the section “Assigning substrates to proteases”.

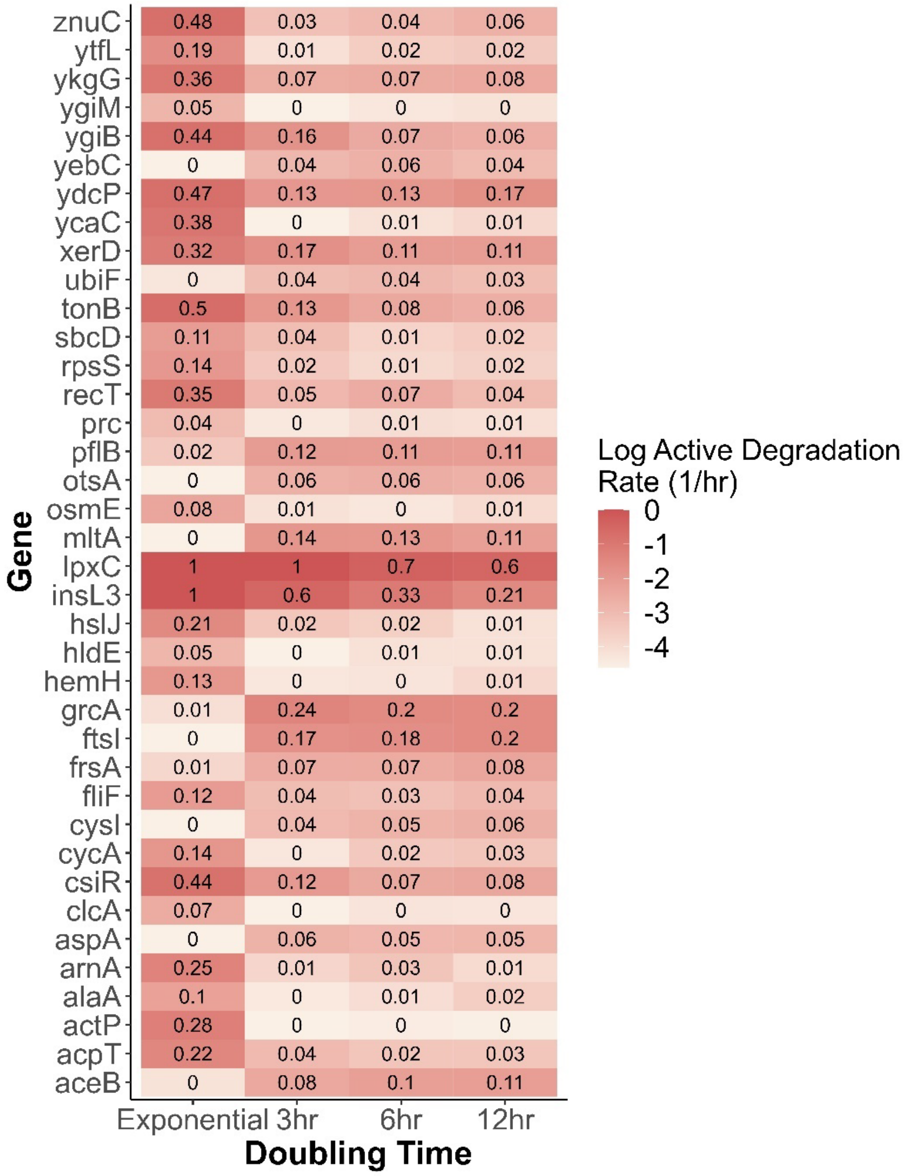

Supplementary Figure 8. Proteins whose active degradation is dependent on growth rate. We tested whether each protein's active degradation rate was correlated with growth rate using Pearson's correlation coefficient. P-values were corrected for multiple hypothesis testing using the q-value package in R. Proteins shown in the heat map above met the significance threshold with a false-discovery rate of 0.01. Text values on each tile are the active degradation rate in units of hr^{-1} and are capped at 1. Proteins were excluded if they did not have a total half-life significantly less than the dilution-only half-life in at least one condition (T-test with a significance threshold of 0.05), or if they did not have an active degradation rate of more than 4% per hour (approximate bulk active degradation) in at least one condition.

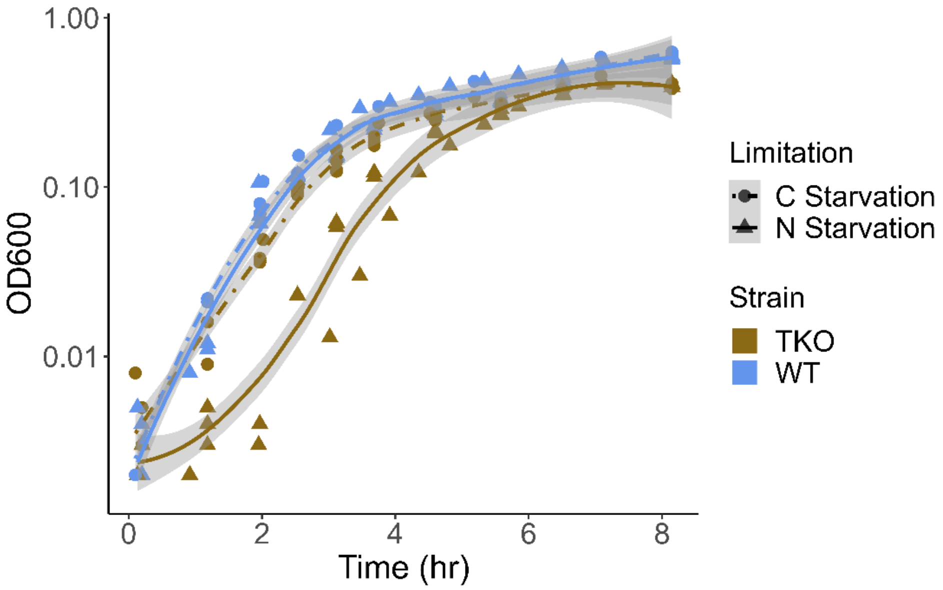

Supplementary Figure 9. A triple protease knock-out strain has delayed growth when shifting from nitrogen starvation to rich media. We measured growth curves following nutrient upshift from starvation in minimal media to LB media for both the wild-type strain and the mutant lacking *lon*, *clpP*, and *hslV*. We find that the triple knock-out (TKO, brown) is less able to adapt to the new conditions and has a significant growth delay (~80 minutes) compared to the wild-type (blue) in nitrogen limitation. When the cells were shifted from carbon starvation to LB, the growth of the triple protease knock-out matches the wild-type strain.

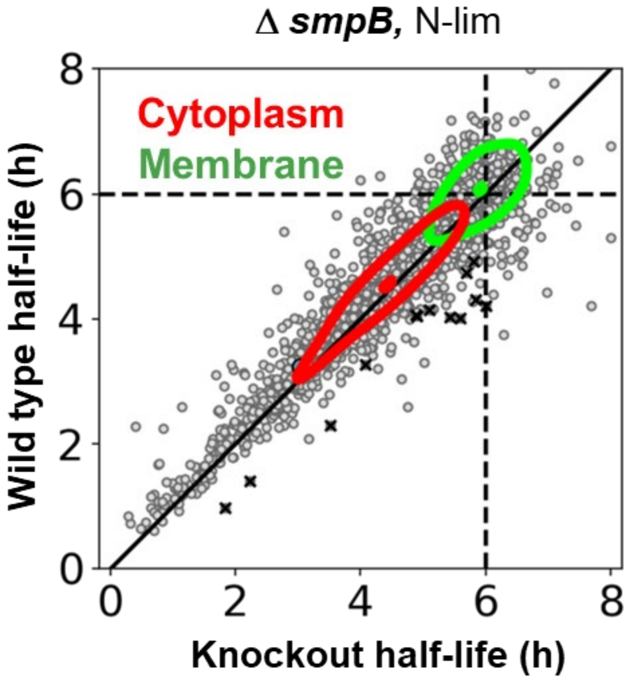

Supplementary Figure 10. Protein turnover rate measurements in a *smpB* knock-out strain. To investigate how much of the observed protein degradation in nitrogen limitation could be attributed to the SsrA tagging system, we knocked out *smpB* in the NCM3722 background strain used throughout this study. SmpB is part of the SsrA tagging complex. We measured gene-by-gene protein turnover in nitrogen limitation with a 6-hour doubling time in duplicate. 12 proteins significantly had a significantly longer total half-life in the mutant strain compared to the wild type (marked with a black *x*): nac, ynfM, gadE, ydhR, moeA, rpsT, pyrI, ybaK, mazF, csrA, truD, yajQ, and ppiC. We did not observe any large-scale change in bulk cytoplasmic protein turnover compared to the wild type.

Supplementary methods

Monoisotopic decay profiles for protein turnover in chemostat

All the variable descriptions (used in the model derivation below) are given in the Supplementary Data 8.

Detailed model

In this section, we will derive the dynamics of M_0, the monoisotopic peak for a peptide with f nitrogen atoms, as a function of time after switching the feed from light to heavy ammonium. The light peak (M_0) decays over time after we switch the media from $^{14}NH_4^+$ to $^{15}NH_4^+$. The mass balance on M_0 is given by:

$$\frac{dM_0}{dt} = \text{Rate of synthesis of } M_0 - \text{Rate of removal of } M_0$$

The rate of synthesis of M_0 is given by the total peptide synthesis rate k_T multiplied by the fraction of that peptide that contributes to the monoisotopic peak. Proteins derive their nitrogen from the amino acid pools N_{AA}, where the fraction of light nitrogen in the amino acid pool is given by $\frac{N_{AAL}}{N_{AA}}$. Since we are only considering the light peak M_0, every nitrogen atom in the peptide) must be light. Hence, k_T is weighted by $\left(\frac{N_{AAL}}{N_{AA}}\right)^f$, where f is the number of nitrogens in the peptide. Finally, k_T is also multiplied by the probability I that all other non-nitrogen atoms in the peptide are light.

$$\text{Rate of synthesis of } M_0 = k_T\left(\frac{N_{AAL}}{N_{AA}}\right)^f I$$

We assume that the degradation of all peptides (including M_0) to follow a first-order decay, originating from the active degradation of the protein with the rate k_D and the dilution of the vessel at the rate D, due to the division of the *E. coli*.

$$\text{Rate of removal of } M_0 = k_D M_0 + DM_0 = (k_D + D)M_0$$

Putting together the two terms, we get,

$$\frac{dM_0}{dt} = k_T\left(\frac{N_{AAL}}{N_{AA}}\right)^f I - (k_D+D)M_0$$

We will now integrate M_0 using the integrating factor $e^{(k_D+D)t}$

$$\int_0^{M_0(t)} d(e^{(k_D+D)t}M_0) = \int_0^t k_T I\left(\frac{N_{AAL}}{N_{AA}}\right)^f e^{(k_D+D)t}dt \qquad (1)$$

In equation 1, k_T is a constant that can be determined by writing the mass balance of the total amount of peptide P. Since the chemostat is at steady state, P does not change with time.

$$\frac{d(P)}{dt} = k_T - (k_D+D)P = 0$$

$$k_T = (k_D+D)P \qquad (2)$$

At time t=0, when we assume there is no heavy nitrogen in the system, M_0 is determined by the natural isotopic abundance of each element.

$$M_0\ (t = 0) = PI \qquad (3)$$

Using equations 1, 2, and 3 we can write the equation for M_0 as follows:

$$\frac{M_0}{PI} = e^{-(k_D+D)t} + (k_D+D)\, e^{-(k_D+D)t} \int_0^t \left(\frac{N_{AAL}}{N_{AA}}\right)^f e^{(k_D+D)t}dt \qquad (4)$$

Next, we determine the time dependence of the fraction of light nitrogen in the amino acid pool $\frac{N_{AAL}}{N_{AA}}$. While the feed is instantaneously switched from $^{14}NH_4^+$ to $^{15}NH_4^+$, there is a delay for nitrogen in amino acids to be exchanged for ^{15}N. Therefore, to determine the composition of M_0, we need to determine the dynamics of $\frac{N_{AAL}}{N_{AA}}$.

As presented in the schematic below, all nitrogen in the system is present in three forms[2]: ammonium in the vessel (N_V), free amino acids in *E. coli* (N_{AA}), and proteins (N_P). Each of these nitrogen pools can be either ^{14}N (L) or ^{15}N (H). Ammonium from the feed (concentration N_F) replenishes ammonium in the vessel (N_V) at the volumetric flow rate of F_0. The ammonium is assimilated by bacteria into amino acids with a specific conversion rate of k_1. Amino acids are also replenished by recycling from degraded proteins at a degradation rate of $k_{d,bulk}$. Nitrogen from amino acids is translated to proteins at a rate k_2. Each form of nitrogen gets depleted from the vessel at a dilution rate, D.

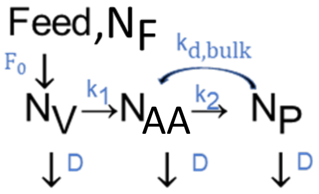

The mass balance of the light nitrogen in amino acids can be written as follows.

$$\frac{d(N_{AAL})}{dt} = k_1 N_{VL} + k_{d,bulk} N_{PL} - k_2 N_{AAL} - DN_{AAL} \qquad (5)$$

The dynamics of N_{AAL} depend on N_{VL} and N_{PL} which are also both time-dependent. Hence, we need to determine their expressions to be able to calculate the dynamics of N_{AAL}. The mass balance for N_{VL} is:

$$\frac{d(N_{VL})}{dt} = N_{FL} F_0 - k_1 N_{VL} - DN_{VL}$$

Since the feed is instantaneously switched to heavy nitrogen, $N_{FL}(t = 0) = 0$. Therefore, the above equation can be simplified.

$$\frac{d(N_{VL})}{dt} = -(k_1 + D)\, N_{VL}$$

Integrating and using the boundary condition $N_{VL}(t = 0) = N_V$

$$N_{VL} = N_V\, e^{-(k_1 + D)\, t} \qquad (6)$$

Similarly, the mass balance of light nitrogen in proteins is:

$$\frac{d(N_{PL})}{dt} = k_2 N_{AAL} - k_{d,bulk} N_{PL} - DN_{PL} \qquad (7)$$

Simultaneously solving equation 5, 6, 7 will give us the desired equation for N_{AAL}. But the above equations involve the undetermined rate parameters F_0, k_1, k_2, and $k_{d,bulk}$. These parameters need to be determined to be able to solve the system.

At steady state, when nitrogen fluxes are balanced, the labeling kinetics can be written down such that only the pool sizes are relevant instead of the rate parameters F_0, k_1, k_2, and $k_{d,bulk}$. It is easier to estimate the pool sizes instead of the rate parameters. Using the mass balance on total pools (heavy plus the light) of all the nitrogen forms, we will rewrite the equations 5, 6, 7.

$$\text{Let } N = N_{AA} + N_P + N_V.$$

At steady state, total balance on N gives us:

$$N_F F_0 = DN \tag{8}$$

At steady state, total balance on N_V gives us:

$$N_F F_0 = k_1 N_V + DN_V \tag{9}$$

Similarly, at steady state, total balance on N_P gives us:

$$k_2 N_{AA} = k_{d,bulk} N_P + DN_P \tag{10}$$

Let us define some parameters in the form of pool sizes:

$$c = N_P/N_{AA} \ ; \ d = N_V/N_{AA} \ ; \ a = (k_{d,bulk} + D)/D$$

Using equations 8, 9, 10 and the definition of c, d, and a:

$$k_1 = D(c+1)/d \tag{11}$$

$$k_2 = Dac \tag{12}$$

Using equations 5, 6, 11, and 12, the dynamics of N_{AAL} can now be written as:

$$\frac{d(N_{AAL})}{dt} = k_1 N_V \, e^{-(k_1+D)\,t} + D(a-1)N_{PL} - D(ac+1)N_{AAL} \tag{13}$$

Using equations 7, 11, and 12, the dynamics of N_{PL} can now be written as:

$$\frac{d(N_{PL})}{dt} = DacN_{AAL} - DaN_{PL} \tag{14}$$

Equations 13 and 14 form a coupled system of equations that is analytically solvable. The box below represents the details of solution for N_{AAL}.

Solving the coupled system of equations 13 and 14 to get the dynamics of N_{AAL}

$$\frac{d(N_{AAL})}{dt} = k_1 N_V \, e^{-(k_1+D)\,t} + D(a-1)N_{PL} - D(ac+1)N_{AAL} \tag{13}$$

$$\frac{d(N_{PL})}{dt} = DacN_{AAL} - DaN_{PL} \tag{14}$$

First solve the homogenous part of the equation. The matrix of interest is

$$\begin{pmatrix} -D(ac+1) & D(a-1) \\ Dac & -Da \end{pmatrix}$$

Eigen values and the corresponding eigen vectors are:

$$\lambda_1 = -D, v_1 = \begin{bmatrix} 1 \\ ac/(a-1) \end{bmatrix}; \lambda_2 = -Da(c+1), v_2 = \begin{bmatrix} 1 \\ -1 \end{bmatrix}$$

$$N_{AAL} = M_1 e^{-Da(c+1)t} + M_2 e^{-Dt} + M_3 e^{-(k_1+D)\,t}$$

$$N_{PL} = -M_1 e^{-Da(c+1)t} + \frac{M_2 ace^{-Dt}}{a-1}$$

Using boundary conditions, $N_{AAL}(t=0) = N_{AA}$ and $N_{PL}(t=0) = N_P$, we can determine the values of M_1 and M_2 using the boundary conditions. M_3 can be determined using the method of undetermined coefficients.

Finally,

$$M_3 = \frac{N_{AA}\,(c+1)}{dac-c-1};\; M_2 = \left(\frac{N_{AA}\,(c+1)(a-1)}{ac+a-1}\right)\left(\frac{dac-c-2}{dac-c-1}\right);\; M_1 = N_{AA} - M_3 - M_2$$

$$N_{AAL} = M_1 e^{-Da(c+1)t} + M_2 e^{-Dt} + M_3 e^{-(k_1+D)\,t} \tag{15}$$

Equations 4 and 15 together represent the coupled system we need to solve. In order to solve the above system of equations, we need to estimate the parameters:

$$c = N_P/N_{AA}\;;\; d = N_V/N_{AA}\;;\; a = (k_{d,bulk} + D)/D$$

The supplementary table 1 details how each parameter was initially estimated. A sensitivity analysis follows in the next section to determine the effect that errors in our estimates have on the dynamics of N_{AAL} and M_0.

Supplementary table 1: Model parameters and their estimation criteria.

Parameter	Estimation
$c = N_P/N_{AA}$	Determined using the literature values of pool sizes [3]. The nitrogen present in the form of proteins (N_P) is ~ 1000 mM, and the pool size of nitrogen in the form of free amino acids N_{AA} is ~150 mM.<br>=> 1000/ 150 = 6.67.<br><br>The range of c is [1, ∞)
$d = N_V/N_{AA}$	For N-lim, this parameter is approximately 0 because all the nitrogen in the vessel is taken up by the bacteria.

	For the P-lim and C-lim conditions, we estimate this quantity as follows:<br><br>The concentration of nitrogen in the feed for P-lim and C-lim chemostats is 9.5 mM and for N-lim chemostats is 1.9 mM. Under N-lim, the cells consume all the nitrogen provided and grow to approximately the same OD_{600} as in P and C-lim. Therefore, we assume that the amount of nitrogen consumed by the bacteria in P-lim and C-lim compared to the total nitrogen in the vessel, are in the ratio of 1.9:9.5<br><br>i.e., $\frac{1.9}{9.5} = \frac{N_P+N_{AA}}{N_P+N_{AA}+N_V}$. Using this equation and the ratio of c = N_P/N_{AA}, d comes out to 30.67.<br><br>The range of d is [0, ∞)
$a=(k_{d,bulk}+D)/D$	For P-lim and C-lim, since most of the proteins are stable, it is assumed that the recycling flux is negligible: $k_{d,bulk}=0$, hence a=1<br><br>For N-lim, Fig. 3G shows the percentage of active turnover per hour calculated by multiplying the molecular weight and its abundance to the active half-life calculated using the simplified model (detailed in the subsequent section). The exact formula is presented in the supplement under the section "Percentage of active proteome turnover per unit hour". In N-lim, 4.5% of the proteome is actively turned over each hour.<br><br>a= (1+0.045/(log(2)/6))= 1.89<br><br>The range of a is [1, ∞)

Given these parameter estimates, under N-lim, since the pools of nitrogen outside the cell and inside the vessel are 0, $N_V \rightarrow 0$ and equation 13 simplifies to

$$\frac{d(N_{AAL})}{dt} = D(a-1)N_{PL} - D(ac+1)N_{AAL}$$

This simplifies the solution, equation 15, by eliminating the third term with M_3.

$$N_{AAL}=M_1e^{-Da(c+1)t} + M_2e^{-Dt} \quad (16)$$

Therefore, the equation 15 represents the dynamics of N_{AAL} for nitrogen rich P-lim and C-lim conditions whereas the equation 16 represents the dynamic of N_{AAL} in N-lim conditions.

Sensitivity analysis

In this section we compare the dynamics of N_{AAL}, $N_{AAL}{}^{f}$, M_0 for different values of c, d and a under N-lim and C-lim/P-lim. The analysis is divided into 4 cases:

Case 1: Model P-lim/C-lim such that $N_V \neq 0$, for a peptide which is not actively degrading such that :-> $k_D = 0$, f = 8, and D = log(2)/6

Case 2: Model P-lim/C-lim such that $N_V \neq 0$, for a peptide which is actively degrading with the active half-life of 1 hour, such that :-> k_D = log(2)/1, f = 8, and D = log(2)/6

Case 3: Model N-lim such that $N_V = 0$, for a peptide which is only diluting such that :-> $k_D = 0$, f = 8, and D = log(2)/6

Case 4: Model N-lim such that $N_V = 0$, for a peptide which is actively degrading such that :-> k_D = log(2)/6, f = 8, and D = log(2)/6

For each case, we plot the solutions for $N_{AAL}, {N_{AAL}}^f, M_0$ for the estimated values of c, d, and a along with the solutions when each parameter is changed by an order of magnitude. We excluded values which are outside the possible range for each parameter. Finally, we plot the solutions when all parameters are changed by an order of magnitude simultaneously.

Supplementary figure 3, shows the profile of three quantities, $N_{AAL}, {N_{AAL}}^f, M_0$ with respect to time for different ranges of c, d, a. These plots show that N_{AAL} is sensitive to the exact choice of parameter values. However, since the dynamics of N_{AAL} only affect M_0 through ${N_{AAL}}^f$, M_0 is insensitive to the exact parameter values.

For a shotgun proteomics experiment, tryptic peptides must have at least seven amino acids to be considered. Since tryptic peptides end in the amino acids R or K, each peptide will have a minimum of eight nitrogen atoms. As the number of nitrogen atoms in a peptide increases, the impact of N_{AAL} dynamics on M_0 decreases. Hence, the above analysis is conducted for peptides with eight nitrogen atoms. Even for this length, the M_0 dynamics are not impacted by the changes in parameter values.

Simplified model

Given that the exact profile of N_{AAL} has little impact on M_0, we propose that the dynamics of nitrogen available to translate protein can be simplified to an exponential with one parameter. This makes the integration analytical and simplifies the fitting procedure.

$$\frac{N_L}{N_T} = e^{-DKt}$$

where K ∈ [1, ∞}. K determines how fast the nitrogen available to make the proteins is exchanged.

Balance on the monoisotopic peak (M_0) for the simplified model:

$$\frac{d(M_0)}{dt} = \text{Rate of synthesis of } M_0 - \text{Rate of removal of } M_0$$

As for the case of detailed model,

The rate of synthesis of M_0 is given by the total peptide synthesis rate (k_T) multiplied by the fraction of that peptide that contributes to the monoisotopic peak. Finally, this quantity is also multiplied by the probability that all other non-nitrogen atoms are light in the peptide (I).

$$\text{Rate of synthesis of } M_0 = k_T \left(\frac{N_L}{N_T}\right)^f I$$

We assume that the degradation follows a first-order decay. The peptide is actively degraded with the rate k_D and diluting of the vessel at the rate D.

$$\text{Rate of removal of } M_0 = k_D M_0 + DM_0 = (k_D + D)M_0$$

Putting together the two terms, we get,

$$\frac{d(M_0)}{dt} = k_T \left(\frac{N_L}{N_T}\right)^f I - (k_D+D)M_0$$

We will now integrate M_0 using the integrating factor $e^{(k_D+D)t}$

$$\int_0^{M_0(t)} d(e^{(k_D+D)t}M_0) = \int_0^t k_T I \left(\frac{N_L}{N_T}\right)^f e^{(k_D+D)t} dt \qquad (A1)$$

In equation A1, k_T is a constant that can be determined by writing the mass balance on total peptide P. Since the chemostat is at steady state, the amount of total peptide does not change with time.

$$\frac{d(P)}{dt} = k_T - (k_D+D)P = 0$$

$$k_T = (k_D+D)P \qquad (A2)$$

At time t=0, when we assume there is no heavy nitrogen in the system, M_0 can be entirely determined by the natural isotopic abundance of each element.

$$M_0\ (t = 0) = PI \qquad (A3)$$

Using equations A1, A2, and A3 we can write the equation for M_0 as follows:

$$\frac{M_0}{PI} = e^{-(k_D+D)t} + (k_D+D)\, e^{-(k_D+D)t} \int_0^t \left(\frac{N_L}{N_T}\right)^f e^{(k_D+D)t} dt \qquad (A4)$$

Using the above simplified form of $\frac{N_L}{N_T}$ and integrating it over time,

$$\frac{M_0}{PI} = \frac{(k_D+D)\, e^{-fDKt} - fDK\, e^{-(k_D+D)\, t}}{(k_D+D) - fDK} \qquad (A5)$$

Solving equation A5 requires an estimate for $K \in [1, \infty\}$, which describes how quickly nitrogen is exchanged within the cell. Our initial parameter estimates were K=100 for N-lim cells, since there is little free nitrogen in the system, and K=1 for P-lim and C-lim cells, indicating that most nitrogen is present outside the cells and is only exchanged by dilution out of the vessel and not by bacterial consumption. As seen in the Supplementary figure 4 the M_0 profiles are robust to the exact choice of K, particularly when K is large.

Finally, Supplementary figure 5 shows the equivalence of simplified and detailed model by comparing the M_0 profiles for all three conditions for different k_D. The simplified model is a reasonable approximation for the detailed model.

Protein half-life fitting

The experimentally measured values of M_0 needs to be normalized for pipetting errors and for aligning deviations of the measured chemostat dilution times to desired dilution time (e.g. 5.7, 6.3, or 5.9 hours to 6.0 hours). We assume that the majority of membrane peptides (annotations from Uniprot) are stable in each chemostat condition, i.e., the median experimentally measured M_0 should align with the model predictions for no active turnover ($k_D = 0$). To ensure this, we use a set of "stable membrane peptides" to find a normalizing constant for each time point. The M_0 values for every protein are then divided by their corresponding constant to normalize the data.

To remove membrane peptides which are actively degrading, we applied the k-means clustering algorithm to cluster the peptides according to their M_0 decay profiles over time (Supplementary figure 6). The top panel shows k-means clustered membrane peptide profiles before normalization, for an example P-lim condition. The numbers in the legend for each color represent the number of peptides in each cluster. Most peptides from membrane proteins fall in the purple cluster, which agrees well, but not perfectly, with the theoretical prediction for a protein with k_D=0. All membrane peptides except those that clustered into the most rapidly degrading profiles were used for normalization (in this example, the red cluster in the top plot in Supplementary figure 6).

The correction values for each time point were obtained by minimizing the least square difference between the theoretical value (Eq. A5 with k_D=0) and the median values of the experimentally observed M_0 for all the g groups and 8 time points at once. Gradient descent (curve_fit function in Python) was used to find the minima.

- ⇨ Fractional form of the theoretical M_0 ($\widetilde{M}_{0_{\text{theoretical, g, t}}}$) is given by $\frac{M_{0_{\text{theoretical,g,t}}}}{\sum_t M_{0_{\text{theoretical,g,t}}}}$
- ⇨ Median of the fractional form of the experimental M_0 (mdn $\widetilde{M}_{0_{\text{experimental, g, t}}}$) is given by $\underset{k}{\text{median}} \frac{M_{0_{\text{experimental,g,t,k}}}}{\sum_t M_{0_{\text{experimental,g,t,k}}}}$, where k corresponds to all the peptides in a group g.
- ⇨ a is a vector of length 8 and a_t denotes an element in the vector.

$$\hat{a} \triangleq \arg\min_a \sum_g \sum_{t=0}^{7} \left(\widetilde{M}_{0_{\text{theoretical, g, t}}}(f) - a_t \left(\text{mdn } \widetilde{M}_{0_{\text{experimental, g, t}}}\right)\right)^2$$

Vector $\hat{a}$ contains the normalizing constants for each time point. The M_0 values for each time point of the entire dataset (experimentally measured values of M_0 for all peptides) are then

divided by their corresponding constants to normalize the data. The bottom panel of Supplementary figure 6 shows data for all peptides with 12 nitrogen atoms for the same P-lim condition after normalization. The numbers in the legend for each color represent the number of peptides in each cluster. The peptides were divided into g groups based on the number of nitrogen atoms (f) the constituent peptides contain. To avoid forcing the time 0 data point to the value of 1, we used the fractional form of the theoretical M_0 decay (Eq. A5) by dividing the value at each time point with the sum of values from the 8 time points. Accordingly, the fractional form of experimental data was also obtained.

Once normalized, the data from all peptides of a given protein is fit to obtain a protein-specific k_D. To avoid forcing the value of the first time point to 1, we used the fractional form (Eq. A5) by dividing the experimental value at each time point with the sum of values from the 8 time points. For a protein p with i peptides each containing f_i nitrogen atoms, we construct a theoretical matrix of (i x 8) values. Accordingly, the corresponding matrix of experimental values is created. The k_D for each protein is obtained by minimizing the least square difference between the theoretical value (Eq. A5 with a free parameter $k_D + D$) and the experimentally observed values. Gradient descent (curve_fit function in python) was used to find the minima.

- ⇨ Fractional form of the theoretical M_0: $\widetilde{M0}_{\text{theoretical, p, i, t}}(k_D,f)$ is given by $\frac{M0_{\text{theoretical, p, i, t}}(k_D,f)}{\sum_t M0_{\text{theoretical, p, i, t}}(k_D,f)}$
- ⇨ Fractional form of the experimental M_0: $\widetilde{M0}_{\text{experimental, p, i, t}}$ is given by $\frac{M0_{\text{experimental, p, i, t}}}{\sum_t M0_{\text{experimental, p, i, t}}}$

$$\widehat{k_{D,p} + D} \triangleq \arg\min_{k_{D,p}} \sum_i \sum_{t=0}^{7} \left(\widetilde{M0}_{\text{theoretical, p, i, t}}(k_D,f) - \widetilde{M0}_{\text{experimental, p, i, t}}\right)^2$$

We also assign confidence intervals to the fitted half-lives. Curve- fit function in python along with returning the parameter estimate also returns the variance estimate of the fitted parameter. To get the one standard deviation error on the parameter, we take the square root of the estimated variance. This is the error with 67% confidence interval. To get the 95% CI, we should look up for the Z-table. However, this is only valid if the parameters follow a normal distribution, which is not the case for small sample size. This is where the t-distribution comes in handy. Therefore we evaluate, Percent point function (inverse of cdf) at a value of 0.975 given degrees of freedom (dof= Number of data points – number of estimated parameters = i x8 -1) (Supplementary Data 7)

Next, we want to find proteins that are actively degraded. For bacteria doubling every 6 hours the null hypothesis is H_0: $T_{1/2} = 6$, and the alternative is H_1: $T_{1/2} < 6$ and the test statistic $t = (\bar{x}-6)\Big/\sqrt{\frac{\sigma^2}{2}}$ where $\bar{x}$ is the sample mean and σ^2 is the sample variance calculated using the two replicates. *p*-values are assigned using a one-tailed t-test. We called proteins with a *p*-value < 0.05 actively degrading.

Cumulative fold enrichment calculation

For the molecular weight case the cumulative log enrichment for a particular half-life is defined as:

$$\mathrm{Log2}\left(\frac{\text{\# of small proteins (Mwt < 10 KDa) < half-life}}{\text{\# of large proteins(Mwt} \geq \text{10 KDa)< half-life}} \times \frac{\text{Total \# of large proteins (Mwt} \geq \text{10 KDa)}}{\text{Total \# of small proteins (Mwt < 10 KDa)}}\right)$$

Similarly, for the intrinsically disordered case the cumulative log enrichment for a particular half-life is defined as:

$$\mathrm{Log2}\left(\frac{\text{\# of disordered proteins (}\geq\text{ 50\% disorder) < half-life}}{\text{\# of ordered proteins (< 50\% disorder)< half-life}} \times \frac{\text{Total \# of ordered proteins (< 50\% disorder)}}{\text{Total \# of disordered proteins (}\geq\text{ 50\% disorder)}}\right)$$

Likelihood ratio calculations for the constant and the scaled model

The goal of this section is to determine which of the models, constant vs scaled from Figure 6 fit the data better. To discriminate between the two models, we calculate the likelihood of observing the total $T_{1/2}$ in condition i given the model and its parameters.

Both the constant and the scaled model describe the relationship between $T_{1/2}$ total for bacteria doubling with 12 hours and a shorter doubling time. The index p denotes individual proteins.

<u>Scaled model:</u>

From Fig. 6.

$$\frac{k_{total,p,x=j}}{k_{total,p,x=i}} = \frac{r_{i,j}\, k_{dilution,i} + r_{i,j}\, k_{active,p,i}}{k_{dilution,i} + k_{active,p,i}} = r_{i,j}$$

Let j be the condition where bacteria doubles with 12 hours:

$$\frac{k_{total,p,x=12}}{k_{total,p,x=i}} = r_{i,12}$$

Since $k_{total} = \ln 2/T_{1/2}$

$$T_{1/2,\ p,i} = r_{i,12}\, T_{1/2,p,12}$$

We use regression-based approached to fit the above model to our data by assuming $T_{1/2,\ 12}$ as our independent variable.

$$T_{1/2,\ p,i} = r_{i,12}\, T_{1/2,\ p,12} + \epsilon_{scaled}$$

We assume that ϵ_{scaled} has a Gaussian distribution denoted by N $(0, \sigma_{scaled}^2)$. Therefore, we can write,

$$p\left(\epsilon_{scaled}\ \middle|\ T_{1/2,p,\ i},\ T_{1/2,\ p,12},\ \sigma_{scaled}\right) = N(0|\mu_p{=}(T_{1/2,\ p,i} - r_{i,12}T_{1/2,\ p,12}), \sigma_{scaled}^2)$$

Constant model:

From Fig. 6.

$$\frac{k_{total,p,x=j}}{k_{total,p,x=i}} = \frac{r_{i,j}\, k_{dilution,i} + k_{active,p,i}}{k_{dilution,i} + k_{active,p,i}}$$

Let j be the condition where *E. coli* doubles every 12 hours:

$$\frac{k_{total,p,x=12}}{k_{total,p,x=i}} = \frac{r_{i,12}\, k_{dilution,i} + k_{active,p,i}}{k_{dilution,i} + k_{active,p,i}}$$

Since $k_{total} = \ln 2/T_{1/2}$

$$\frac{T_{1/2,p,i}}{T_{1/2,p,12}} = \frac{r_{i,12}\, k_{dilution,i} + k_{active,p,i}}{k_{dilution,i} + k_{active,p,i}}$$

Because of the complexity of the right-hand side expression, we rewrite the above equation as

$$T_{1/2,\, p,\, i} = g(T_{1/2,p,12})$$

Here, g represents a function that is dependent on $T_{1/2,\, 12}$

We use regression-based approached to fit the above model to our data:

$$T_{1/2,p,i} = g(T_{1/2,p,12}) + \epsilon_{constant}$$

We assume that $\epsilon_{constant}$ has a Gaussian distribution denoted by N $(0, \sigma_{constant}^2)$. Therefore, we can write,

$$p(\epsilon_{constant} \,|\, T_{1/2,p,i}, T_{1/2,p,12}, \sigma_{constant}) = N\,(0 | \mu_p = (T_{1/2,\, p,i} - g(T_{1/2,p,12})), \sigma_{constant}^2)$$

Both the models have an undetermined parameter, $\sigma_{constant}$ and σ_{scaled}. The box below presents a derivation for estimating these parameters.

Below we present a generalized derivation for estimating the value of σ for a regression-based modeling.

Estimating the parameter σ by maximizing the probability functions with respect to it (Maximum likelihood estimator):

If n is the total number of proteins, likelihood (L) is given by:

$$L = \prod_{p=1}^{n} p(0 |\, \mu_p, \sigma^2)$$

$$\text{Log}(L) = \frac{-n}{2}\log(2\pi) - n\log(\sigma) - \frac{1}{2\sigma^2}\sum_{i=1}^{n}\mu_p^2$$

Taking the derivative wrt to σ and equating to zero:

$$\hat{\sigma}^2 = \frac{\sum_{p=1}^{n}\mu_p^2}{n} = \frac{\text{RSS}}{n}$$

The quantity RSS is defined as the residual sum of squares.

Once the $\sigma_{constant}$ and σ_{scaled} have been estimated, they can be substituted into the likelihood functions. As iterated above the quantity of interest that helps determine model that best describes the data is the ratio of likelihoods $\frac{\hat{L}_{CONSTANT}}{\hat{L}_{SCALED}}$.

$$\frac{\hat{L}_{CONSTANT}}{\hat{L}_{SCALED}} = \exp(-n/2(\ln(RSS_{constant}) - \ln(RSS_{scaled})))$$

$$RSS_{constant} = \sum_{p}(T_{1/2,\,i,\,p} - rT_{1/2,\,12,p})^2$$

$$RSS_{scaled} = \sum_{p}(T_{1/2,i,\,p} - g(T_{1/2,\,12,p}))^2$$

where the index p is for the proteins and n is the total number of proteins.

We further calculate the *p*-value using the F-test to compare variances of the two groups.

Assigning substrates to proteases

To identify the proteins that are stabilized on knocking out a protease, we compared the half-lives ($T_{1/2}$) in the wild-type (WT) and the knockout (KO) strain. The null and alternative hypotheses are H_0: $T_{1/2,\,WT} = T_{1/2,\,KO}$, H_1: $T_{1/2,\,WT} < T_{1/2,\,KO}$ and the test statistic $t = (\bar{x}_{WT} - \bar{x}_{KO}) / \sqrt{\frac{\sigma_{WT}^2 + \sigma_{KO}^2}{2}}$ where $\bar{x}$ is the sample mean and σ^2 is the sample variance calculated using replicates. *p*-values are assigned using one-tailed t-tests. We called the proteins with *p*-value < 0.10 confidently stabilized.

For the substrates that are confidently stabilized in the triple KO, we could assign the percentage of contribution towards stability by each of the 6 mutually exclusive categories: ClpP alone, Lon alone, HslV alone, additive contribution, redundant contribution and actively degrading in triple knockout. All the calculations are done on the rates ($k = \ln(2)/T_{1/2}$) and not on half-lives.

Gain in stability by each individual knockout is defined as

$$Dk_{KO} = \begin{cases} k_{WT} - k_{KO} & \text{if } k_{WT} > k_{KO} \\ 0 & \text{otherwise} \end{cases}$$

If D is the bacterial growth rate, percentage contribution of individual knockouts towards complete stability is defined as:

$$\%\ \text{KO}=\frac{Dk_{KO}}{k_{WT}\text{-}D}$$

$$\%\ \text{Additive}=\frac{Dk_{\Delta clpP}+Dk_{\Delta lon}+Dk_{\Delta hslV}}{k_{WT}\text{-}D}$$

$$\%\ \text{Redundant}=\frac{Dk_{\Delta clpP\Delta lon\Delta hslV}-(Dk_{\Delta clpP}+Dk_{\Delta lon}+Dk_{\Delta hslV})}{k_{WT}\text{-}D}$$

$$\%\ \text{Unexplained}=\frac{(k_{WT}\text{-}D)-Dk_{\Delta clpP\Delta lon\Delta hslV}}{k_{WT}\text{-}D}$$

These percentages are further modified so that any negative values are set to 0. The modified percentages are renormalized by dividing them with the sum of all the categories.

We assigned each confidently degrading protein to one of six categories: clpP substrate, lon substrate, hslV substrate, additively degraded by multiple proteases, redundantly degraded by multiple proteases, and unexplained. We assigned each protein using the following criteria in order:

1. Proteins were assigned as a substrate of a single protease if at least 60% of the increase in stability is explained by the corresponding knock-out.

2. If the individual contributions of each protease summed to at least 70%, the protein was categorized as additive.

3. If more than 20% was explained by the redundancy, the proteins were categorized as redundant.

4. All the remaining proteins and the ones that are still short-lived in the Δ*clpP*Δ*lon*Δ*hslV* were categorized as unexplained.

Percentage of active proteome turnover per unit hour

Absolute protein abundances (P_a) are calculated using label free mass spectrometry (Supplementary Data 6). The proteins with half-life greater than the doubling time are called stable and assigned a half-life equal to the doubling time ($T_{1/2,\ cap}$). Percentage of active proteome turnover is calculated as $\frac{\sum_i MW_i \frac{\ln(2)}{T_{1/2,cap,i}} P_{a,i} - \sum_i MW_i\, D\, P_{a,i}}{\sum_i MW_i\, P_{a,i}}$ where i is an index for proteins and D is the chemostat dilution rate and MW is the molecular weight of the proteins.

Batch turnover:

Exponentially growing cells in minimal media are washed and resuspended in nitrogen-depleted media. Proteomics samples are collected after the switch and protein profiles are fitted with exponential curves to obtain the turnover rates.

References

1. Biorender. https://www.biorender.com/.
2. Yuan, J., Fowler, W.U., Kimball, E., Lu, W. & Rabinowitz, J.D. Kinetic flux profiling of nitrogen assimilation in Escherichia coli. *Nat Chem Biol* **2**, 529-530 (2006).
3. Bennett, B.D. et al. Absolute metabolite concentrations and implied enzyme active site occupancy in Escherichia coli. *Nat Chem Biol* **5**, 593-599 (2009).
