# Supplementary material for: Global protein turnover quantification in Escherichia coli reveals cytoplasmic recycling under nitrogen limitation
Source: Nat Commun. 2024 Jul 13;15:5890. doi: 10.1038/s41467-024-49920-8 (PMC11246515; doi:10.1038/s41467-024-49920-8)
Supplement: Supplementary file 3 — Description of Additional Supplementary Files [file 41467_2024_49920_MOESM3_ESM.pdf]

Description of Additional Supplementary Files

File Name: Supplementary Data 1
Description: Half-lives for 3262 proteins across 13 growth conditions

File Name: Supplementary Data 2
Description Assignment of protein substrates to proteases

File Name: Supplementary Data 3
Description: Rapidly degrading proteins

File Name: Supplementary Data 4
Description N terminus residue of proteins

File Name: Supplementary Data 5
Description: Relative protein levels

File Name: Supplementary Data 6
Description: Absolute protein levels

File Name: Supplementary Data 7
Description: 95% Confidence intervals on half-lives for 3262 proteins across 13 growth conditions

File Name: Supplementary Data 8
Description: Glossary of all the variables used in the derivation of monoisotopic peak decay
