# Supplementary material for: Global protein turnover quantification in Escherichia coli reveals cytoplasmic recycling under nitrogen limitation
Source: Nat Commun. 2024 Jul 13;15:5890. doi: 10.1038/s41467-024-49920-8 (PMC11246515; doi:10.1038/s41467-024-49920-8)
Supplement: Supplementary file 12 — Reporting Summary [file 41467_2024_49920_MOESM12_ESM.pdf]

nature portfolio

Corresponding author(s): Martin Wuehr

Last updated by author(s): Jan 16, 2024

Reporting Summary

Nature Portfolio wishes to improve the reproducibility of the work that we publish. This form provides structure for consistency and transparency in reporting. For further information on Nature Portfolio policies, see our Editorial Policies and the Editorial Policy Checklist.

Statistics

For all statistical analyses, confirm that the following items are present in the figure legend, table legend, main text, or Methods section.

n/a	Confirmed	
☐	☒	The exact sample size (*n*) for each experimental group/condition, given as a discrete number and unit of measurement
☐	☒	A statement on whether measurements were taken from distinct samples or whether the same sample was measured repeatedly
☐	☒	The statistical test(s) used AND whether they are one- or two-sided<br>*Only common tests should be described solely by name; describe more complex techniques in the Methods section.*
☒	☐	A description of all covariates tested
☐	☒	A description of any assumptions or corrections, such as tests of normality and adjustment for multiple comparisons
☐	☒	A full description of the statistical parameters including central tendency (e.g. means) or other basic estimates (e.g. regression coefficient) AND variation (e.g. standard deviation) or associated estimates of uncertainty (e.g. confidence intervals)
☐	☒	For null hypothesis testing, the test statistic (e.g. *F*, *t*, *r*) with confidence intervals, effect sizes, degrees of freedom and *P* value noted<br>*Give P values as exact values whenever suitable.*
☒	☐	For Bayesian analysis, information on the choice of priors and Markov chain Monte Carlo settings
☒	☐	For hierarchical and complex designs, identification of the appropriate level for tests and full reporting of outcomes
☐	☒	Estimates of effect sizes (e.g. Cohen's *d*, Pearson's *r*), indicating how they were calculated

Our web collection on statistics for biologists contains articles on many of the points above.

Software and code

Policy information about availability of computer code

Data collection	The code used for analyzing these data sets can be found on our lab’s Github at https://github.com/wuhrlab/ProteinTurnoverEcoli. The code is also citable using: DOI: 10.5281/zenodo.10895828
Data analysis	The code used for analyzing these data sets can be found on our lab’s Github at https://github.com/wuhrlab/ProteinTurnoverEcoli. The code is also citable using: DOI: 10.5281/zenodo.10895828

For manuscripts utilizing custom algorithms or software that are central to the research but not yet described in published literature, software must be made available to editors and reviewers. We strongly encourage code deposition in a community repository (e.g. GitHub). See the Nature Portfolio guidelines for submitting code & software for further information.

Data

Policy information about availability of data

All manuscripts must include a data availability statement. This statement should provide the following information, where applicable:

- Accession codes, unique identifiers, or web links for publicly available datasets
- A description of any restrictions on data availability
- For clinical datasets or third party data, please ensure that the statement adheres to our policy

The mass spectrometry proteomics data have been deposited to the ProteomeXchange Consortium via the PRIDE15 partner repository with the dataset identifier PXD042444.

Research involving human participants, their data, or biological material

Policy information about studies with human participants or human data. See also policy information about sex, gender (identity/presentation), and sexual orientation and race, ethnicity and racism.

Reporting on sex and gender	N/A
Reporting on race, ethnicity, or other socially relevant groupings	N/A
Population characteristics	N/A
Recruitment	N/A
Ethics oversight	N/A

Note that full information on the approval of the study protocol must also be provided in the manuscript.

Field-specific reporting

Please select the one below that is the best fit for your research. If you are not sure, read the appropriate sections before making your selection.

☒ Life sciences ☐ Behavioural & social sciences ☐ Ecological, evolutionary & environmental sciences

For a reference copy of the document with all sections, see nature.com/documents/nr-reporting-summary-flat.pdf

Life sciences study design

All studies must disclose on these points even when the disclosure is negative.

Sample size	All protein turnover rate measurements were replicated for each strain and condition. After collecting replicates for the wild-type strain in carbon limitation at a 6-hour doubling time, we determined that the measurements were highly reproducible and that a significant fraction of the proteome could be confidently assigned as degrading more than dilution at a p-value cutoff of 0.05 (Figure 1E and Figure 2B). The batch starvation assay was also initially done in duplicates. Due to the large sample of the technical replication (>1000 proteins quantified at a false discovery rate of 0.5%), we were able to confidently separate the decrease in protein abundance of the cytoplasmic and membrane-bound proteins with a p-value<1E-15 (Figure 2E). Growth assay comparison of the wild-type strain (NCM3722) and the triple protease knock-out on minimal media with thymidine as the sole nitrogen source were done in triplicate. All triplicates agreed well with each other so no further replication was deemed to be necessary (p-value<1E-4, Figure 5G).
Data exclusions	No data was excluded from analyses.
Replication	All protein turnover rate measurements were replicated for each strain and condition. Each of the 13 turnover experiments were performed in duplicates. These are biological replicates measures months apart.
Randomization	Not relevant to this study - there are no experimental groups.
Blinding	Not relevant to this study - there are no experimental groups.

Reporting for specific materials, systems and methods

We require information from authors about some types of materials, experimental systems and methods used in many studies. Here, indicate whether each material, system or method listed is relevant to your study. If you are not sure if a list item applies to your research, read the appropriate section before selecting a response.

Materials & experimental systems

n/a	Involved in the study
☒	☐ Antibodies
☒	☐ Eukaryotic cell lines
☒	☐ Palaeontology and archaeology
☒	☐ Animals and other organisms
☒	☐ Clinical data
☒	☐ Dual use research of concern
☒	☐ Plants

Methods

n/a	Involved in the study
☒	☐ ChIP-seq
☒	☐ Flow cytometry
☒	☐ MRI-based neuroimaging

Plants

Seed stocks	*Report on the source of all seed stocks or other plant material used. If applicable, state the seed stock centre and catalogue number. If plant specimens were collected from the field, describe the collection location, date and sampling procedures.*
Novel plant genotypes	*Describe the methods by which all novel plant genotypes were produced. This includes those generated by transgenic approaches, gene editing, chemical/radiation-based mutagenesis and hybridization. For transgenic lines, describe the transformation method, the number of independent lines analyzed and the generation upon which experiments were performed. For gene-edited lines, describe the editor used, the endogenous sequence targeted for editing, the targeting guide RNA sequence (if applicable) and how the editor was applied.*
Authentication	*Describe any authentication procedures for each seed stock used or novel genotype generated. Describe any experiments used to assess the effect of a mutation and, where applicable, how potential secondary effects (e.g. second site T-DNA insertions, mosiacism, off-target gene editing) were examined.*
